# Cortical thickness in human V1 associated with central vision loss

**DOI:** 10.1038/srep23268

**Published:** 2016-03-24

**Authors:** Wesley K. Burge, Joseph C. Griffis, Rodolphe Nenert, Abdurahman Elkhetali, Dawn K. DeCarlo, Lawrence W. ver Hoef, Lesley A. Ross, Kristina M. Visscher

**Affiliations:** 1 University of Alabama at Birmingham Department of Psychology, Campbell Hall 415, 1530 3rd Avenue South, Birmingham, AL 35294, USA; 2 University of Alabama at Birmingham School of Medicine Department of Neurology, SC 350, 1720 2nd Ave South Birmingham, AL 35294, USA; 3 University of Alabama at Birmingham School of Medicine Department of Neurobiology, SHEL 911, 1720 2nd Avenue South, Birmingham, AL 35294, USA; 4 University of Alabama at Birmingham School of Medicine Department of Ophthalmology, 700 S. 18th Street, Suite 601, Birmingham, AL 35294, USA; 5 The Pennsylvania State University, Department of Human Development and Family Studies, 119 Health and Human Development Bldg, University Park, PA 16802, USA.

## Abstract

Better understanding of the extent and scope of visual cortex plasticity following central vision loss is essential both for clarifying the mechanisms of brain plasticity and for future development of interventions to retain or restore visual function. This study investigated structural differences in primary visual cortex between normally-sighted controls and participants with central vision loss due to macular degeneration (MD). Ten participants with MD and ten age-, gender-, and education-matched controls with normal vision were included. The thickness of primary visual cortex was assessed using T1-weighted anatomical scans, and central and peripheral cortical regions were carefully compared between well-characterized participants with MD and controls. Results suggest that, compared to controls, participants with MD had significantly thinner cortex in typically centrally-responsive primary visual cortex – the region of cortex that normally receives visual input from the damaged area of the retina. Conversely, peripherally-responsive primary visual cortex demonstrated significantly increased cortical thickness relative to controls. These results suggest that central vision loss may give rise to cortical thinning, while in the same group of people, compensatory recruitment of spared peripheral vision may give rise to cortical thickening. This work furthers our understanding of neural plasticity in the context of adult vision loss.

Macular degeneration (MD) is a disease responsible for over 50% of all visual impairments in the United States^1^. MD causes central retinal lesions, resulting in a loss of central vision essential for everyday activities that require high visual acuity, for example reading or recognizing faces^2^. Fortunately, although this central vision loss can be devastating for MD patients, many learn to rely on their spared peripheral vision to compensate for central vision loss to perform everyday activities^3^. However, there is great variability among MD patients in their ability to compensate for the loss of vision in a part of the visual field (called a scotoma). While there appears to be a trend for larger central scotomas to result in poorer visual function, the characteristics of the scotoma and resulting deficits in acuity do not correlate reliably with metrics of visual function such as reading, visual search, mobility, and activities of daily living^2^,^4^,^5^,^6^. Thus, acuity is not a good metric to assess level of functional vision. Importantly, this suggests that the compensation for loss of vision after MD depends on structures beyond the level of the retina. Clearly, although the behavioral effects of MD appear to depend in part on neural plasticity following vision loss, the impact of MD on the brain is not entirely understood^7^,^8^,^9^,^10^.

Changes in cortical structure are known to occur following drastic changes in visual input in adult animal models^11^,^12^; however, there has been limited evidence of such changes in adult humans. Understanding the degree to which cortical structure can be modified by experience in the adult human brain is essential to the neuroscience and neural plasticity community. Patients with dense central scotomas due to MD are an ideal group to study because they have regions of vision loss as well as regions of spared peripheral vision upon which patients rely more than controls do. Relative to controls, patients with MD have decreased grey matter volume and density in the lesion projection zone – the region of primary visual cortex that receives input from the now damaged macula^13^,^14^,^15^.This suggests cortical atrophy resulting from loss of visual input. However, interpretation of this finding is difficult because it is not known what aspects of cortical anatomy are reflected by measurements of grey matter density and volume. For example, it is unknown whether these measurements reflect changes in cortical thickness, cortical area, or gyrification patterns^16^,^17^,^18^. Further, little attention has been given to the question of whether *increases* in the use of parts of the visual field lead to changes in the structure of early visual areas. This is the first study, to our knowledge, that addresses the degree to which *increased* use of a visual region leads to increases on cortical thickness.

This study compared the cortical thickness of primary visual cortex (V1) between participants with MD who have central vision loss but intact peripheral vision and matched (age, gender, education) normally sighted control participants. Our experimental design allowed us to examine, in the same group of carefully-chosen and rare participants who have dense central vision loss in both eyes, the consequences of both increased and decreased use of a visual field. We hypothesized that participants with MD, as compared to the matched controls, would (a) have thinner cortex in centrally responsive parts of V1 (e.g. lesion projection zone) following the reduced use of central vision, and conversely (b) thicker cortex in peripherally responsive parts of V1 following the increased use of peripheral vision as a compensatory strategy for central vision loss.

## Materials and Methods

### Participants

The University of Alabama at Birmingham (UAB) Institutional Review Board approved this study, and all participants provided informed consent for their participation. All methods were carried out in accordance to the approved guidelines. We recruited ten participants with MD (six females and four males; mean age 63.1 years, range 34–81 years; mean education 14.8, range 3–18 years; see Table 1) and ten control participants with normal vision (minimum visual acuity: 20/44 best eye) matched to each MD participant for age (within five years), gender, and education level (no high school degree, high school degree, some college, college, or advanced degree). The groups were not significantly different in age (T(18) = 0.1103, p = 0.913). Eligibility criteria required that MD participants had been diagnosed with MD in both eyes for at least 2 years and did not suffer from any neurological disorder. The requirement of central vision loss in both eyes can make this a challenging population to recruit. Within the MD group, three participants had juvenile-onset MD (Stargardt disease), and seven MD participants had age-related MD. All MD participants had significant central visual field loss as measured by retinal microperimetry^6^ at a hospital-affiliated clinical center for low vision.

**Figure 1:**
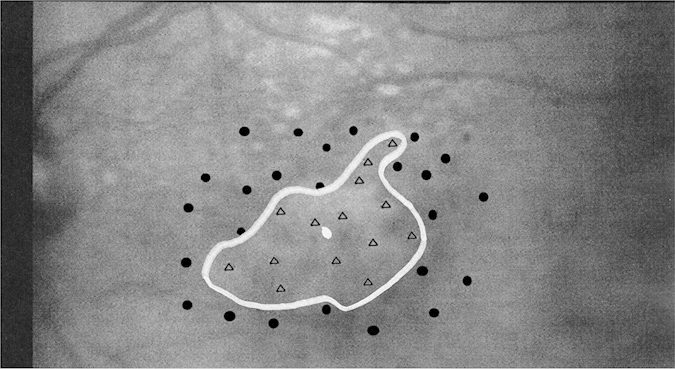
Example Scanning Laser Ophthalmoscope (SLO) image of a macular degeneration participant’s left retina. The black dots indicate visual stimuli that the participant was able to respond to. The black triangles indicate stimuli that the participant did not respond to. The silver dot in the center indicates the likely fovea location. The silver line indicates the extent of the scotoma as drawn by the clinician and author DKD. Measurements of scotoma size were calculated on a subject-by-subject basis using each individual’s SLO image of their retina. A measurement of the diameter was made horizontally through the fovea as well as vertically through the fovea. This measurement was then converted to visual degrees using previously reported methods^20^.

Prior to MRI scanning, participants with MD underwent visual acuity testing (ETDRS)^19^, optical coherence tomography, and retinal microperimetry using the Rodenstock Scanning Laser Ophthalmoscope (SLO). The SLO confirmed that all participants with MD had significant central visual field loss. Each participant’s scotoma was at least 3 degrees visual angle wide as determined by the SLO. The scotoma extent for each participant was hand drawn on each SLO by a trained clinician (Author DKD, See Fig. 1 for an example SLO image) and the extent of the scotoma for each eye was calculated from this hand drawn measurement (Table 1). Measurements of scotoma size were calculated on a participant-by-participant basis using each individual’s SLO image of their retina. We made a measurement of the diameter of the scotoma horizontally through the fovea as well as vertically through the fovea. We converted this measurement to degrees visual angle using previously reported methods^20^. Table 1 provides the mean diameter in visual angle of the scotoma in each eye and is consistent with previous literature^13^,^15^. Each cortical hemisphere processes one half the visual field, therefore, to determine the scotoma’s cortical representation, we used the radius of the scotoma instead of the diameter of the scotoma, and because the cortex receives input from both eyes, the minimum for left vs. right eye was used to determine scotoma extent as used in later analyses.

### Anatomical Scan

A 3 Tesla head-only Siemens Magnetom Allegra was used to acquire a single 3D high-resolution anatomical scan (MPRAGE; T1-weighted; repetition time (TR) = 2250 ms; echo time (TE) = 2.6 ms; field of view [FOV(ap,fh,rl)] = 240 X 256 X 176 mm; slice gap, 0; 1.0 X 1.0 X 1.1 mm voxel size; flip angle (FA) = 10).

### Cortical Reconstruction

Cortical thickness and grey matter volume were calculated using Freesurfer (version 5.3.0) –a surface based analysis tool that calculates the distance between the grey/white matter boundary and the pial surface^21^,^22^,^23^. All regions of interest (ROIs) were created in Freesurfer (version 5.3.0).

### Bar ROIs

**Figure 2:**
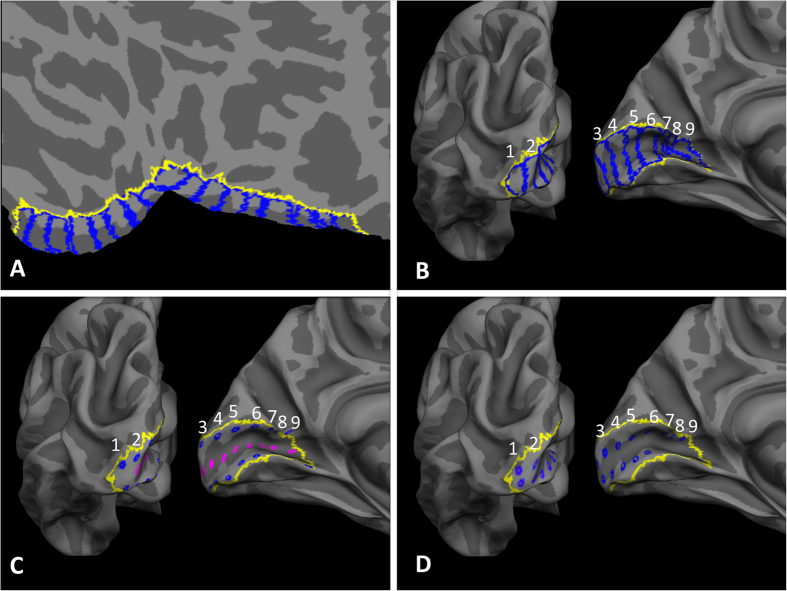
V1 regions of interest. (**A**) Flat-map of an inflated left hemisphere. The yellow line indicates the V1/V2 border as defined by the Freesurfer V1 label. The blue lines indicate the individual labels used for analysis. (**B**) The labels from part A, shown on the fsaverage partially inflated surface. The occipital pole and medial surface are shown. The numbers indicate the ROI naming scheme. (**C**) The gyrus peak (Blue) and depth of sulcus (Magenta) regions of interest on the fsaverage partially inflated surface. (**D**) The bank of the sulcus regions of interest on the fsaverage partially inflated surface.

We created a set (9 per hemisphere, 18 total) of ROIs in V1 of varying eccentricity on a flat-map of the occipital pole using the Freesurfer *fsaverage* brain. These ROIs were defined as bars that extended across the dorsal-ventral axis of V1, perpendicular to the calcarine sulcus (Fig. 2A,B), and correspond to the left or right half of an annulus in the visual field. We hand drew eight such bar ROIs along the calcarine sulcus using the Freesurfer V1 label file (the yellow line in Fig. 2) as a guide. Each ROI had an approximate width of 10 mm as calculated with the *plot_curv* function in *tksurfer*. We also created a ninth ROI consisting of the remaining vertices between the eighth ROI and the end of the V1 label file (Fig. 2A,B). These regions spanned from the V1/V2 border on the inferior gyrus across the depth of the calcarine sulcus to the V1/V2 border on the superior gyrus. These regions therefore span the upper, middle, and lower visual field representations in V1^24^. We hand drew these 9 ROIs for both the left and right hemisphere (total of 18 ROIs) on the Freesurfer *fsaverage* brain, and then using an automated process transformed the ROIs from the *fsaverage* space to each participant’s anatomical space. This approach allows creation of consistent regions across participants. These regions are labeled 1 through 9 in Fig. 2 and each region’s surface area is 309 mm^2^, on average. Published data^25^ gives an estimate of the mean eccentricities of regions 2 to 9, which are: bar #2: 1.34 degrees, bar #3: 2.2 degrees, bar #4: 4.1 degrees, bar #5: 7.3 degrees, bar #6: 14.1 degrees, bar #7: 25.5 degrees, bar #8: 40.0 degrees, and bar #9: 63.3 degrees. The mean eccentricity of bar #1 is likely to be less than 1 degree visual angle, but the published retinotopic data^25^ does not go below 1 degree of visual angle.

### Additional circle ROIs specific to locations along the gyrus and sulcus

Throughout the cortex, the depth of the sulcus is generally thinner than at the gyral crowns^23^. Therefore, any results from the ROIs presented above could be driven by a difference in proportion of gyrus and sulcus represented in any one ROI. To control for the possibility that the proportion of surface area from the gyrus compared to the sulcus could influence our results, we performed the same tests in a separate set of ROIs where we separated gyral and sulcal regions. We created these ROIs as “circles” in V1 (Fig. 2C,D), and each ROI corresponds to a small patch in the visual field at different eccentricities. We used three different locations in the calcarine sulcus to define a new set of ROIs on the *fsaverage* partially inflated surface: 1) Circle ROIs in the gyrus crown: located on the V1 side of the V1/V2 border for both the upper and lower banks of the calcarine sulcus (Fig. 2C, blue), 2) Circle ROIs in the bank of the sulcus: halfway between the gyrus crown and the depth of the sulcus for both the upper and lower banks of the calcarine sulcus (Figs 2D, blue), and 3) Circle ROIs in the depth of sulcus (Fig. 2C, magenta). We used the initial set of ROIs constructed on the flat-map as guides to space these new ROIs approximately 10 mm apart. We created each ROI using Freesurfer as follows: we selected a vertex by hand based on the initial set of ROIs and converted the vertex to a Freesurfer ‘label.’ This single vertex was expanded using the FreeSurfer “Dilate Label” function, which expands the region to include the original vertex and all neighboring vertices. This process of dilation was repeated for each region a total of three times and the identical dilation procedure was repeated with each of the predefined ROIs. This created the regions as shown in Fig. 2C,D. The area of each of these regions was roughly 20 mm^2^. We created the ROIs on both hemispheres, and then transformed the ROIs to each subject’s anatomical space.

### Data Analysis

All MD participants had central vision loss including a minimum diameter of three degrees visual angle (see Participants section). ROIs one through three corresponded approximately to the central 3 degrees visual angle, according to published retinotopic mapping data^25^. Thus, the ROIs one through three are likely to correspond to the regions of vision loss in our MD participants. ROIs four and five correspond to a mean eccentricity of about 4 and 7 degrees, respectively, according to published retinotopic mapping data^25^. These ROIs corresponded generally to the border between the scotoma and healthy retinal tissue. ROIs six through nine corresponded to mean eccentricities of 14 to 63 degrees, and represented the mid to far periphery^25^.

**Figure 3:**
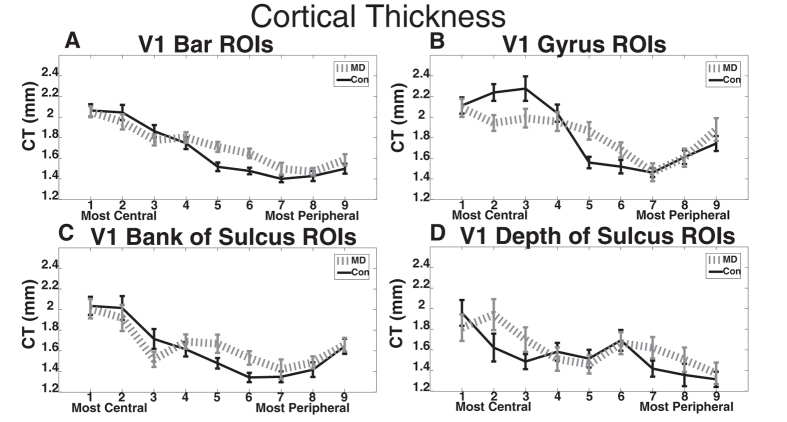
(**A**) Cortical thickness of macular degeneration (MD) and Control participants in the Bar ROIs shown in Fig. 2B. (**B**) Cortical thickness of MD and Control participants in the Circle ROIs shown in Fig. 2C, comprising the gyrus peak along the calcarine sulcus. (**C**) Cortical thickness of MD and Control participants in the Circle ROIs show in Fig. 2D, comprising the bank of the calcarine sulcus. (**D**) Cortical thickness of MD and Control participants in the Circle ROIs shown in Fig. 2C, comprising the depth of the calcarine sulcus. Error bars show standard error of the mean.

Each of the presented analyses used data that were averaged across both hemispheres for each ROI. Similarly, data from the upper and lower bank ROIs (the gyrus and bank of sulcus ROIs from Fig. 2C,D) were averaged together. For each set of regions, we performed two-way mixed-measures ANOVA with a between-subjects factor of group (2 levels) and a within-subjects factor of ROI (ROIs from Fig. 2B; 9 levels). This analysis was chosen as measurements of cortical thickness across V1 were assumed to be dependent samples. We followed up any significant interaction with post-hoc t-tests. Figure 3 shows cortical thickness results across central to peripheral eccentricities in V1.

Previous research has found a decrease in grey matter volume in the lesion projection zone in patients with central vision loss^13^,^14^,^15^. Therefore, in order to directly compare our data to these previous results, we investigated grey matter volume in our participant group using the bar ROIs created for the cortical thickness analysis. For this analysis we used the Freesurfer estimated grey matter volume^26^. Previous papers used voxel-based techniques^13^,^14^,^15^, which are similar in concept but not entirely identical to our technique. We used Freesurfer instead of a voxel-based technique because we could use the same ROIs that we used for cortical thickness analysis, making comparison straightforward.

We first present the results from the Bar ROIs that represent the upper, middle, and lower visual fields for a given eccentricity. Following this we present the results from the additional “circle” ROIs that are specific to a location along the gyrus or sulcus, to control for differences in sulcus/gyrus surface area ratios between groups in a particular ROI. We then present the results of an analysis of cortical thickness relative to the scotoma border. Finally we conclude with the results of the Freesurfer volume based analysis to align our results with previous volume based experiments^13^,^14^,^15^.

## Results

### Cortical Thickness

#### Bar ROI

We performed a two-way mixed-measures ANOVA on data from the bar ROIs with a between-subjects factor of group (2 levels) and a within-subjects factor of ROI (ROIs from Fig. 2B; 9 levels). These data violated the repeated measures ANOVA assumption of sphericity with a Huynh-Feldt Epsilon = 0.77. The data are reported with the corrected degrees of freedom from the Huynh-Feldt Epsilon, an approach appropriate to use with Epsilon greater than 0.75^27^. We found no main effect of group (F(1,18) = 0.76, p = 0.40), a main effect of ROI (F(6.2,110.9) = 44.88, p < .001), and a significant interaction of group by ROI (F(6.2,110.9) = 2.204, p = 0.046). To follow up this significant interaction, we performed independent sample t-tests. We found that the 5^th^ (T(18) = 2.36, p = 0.03) and 6^th^ (T(18) = 2.65, p = 0.02) ROIs were significantly thicker in the MD group compared to the control group. No other post hoc t-test with this set of ROIs was significant (Fig. 3A).

#### Circle ROI - crown of gyrus

We performed a two-way mixed-measures ANOVA on data from the crown of the gyrus ROIs with a between-subjects factor of group (2 levels) and within-subjects factor of ROI (ROIs from Fig. 2C blue; 9 levels). These data violated the repeated measures ANOVA assumption of sphericity with a Huynh-Feldt Epsilon = 0.84. The data are reported with the corrected degrees of freedom. There was no main effect of group (F(1,18) = 0.02, p = 0.90), but there was a main effect of ROI (F(6.8,121.5) = 18.07, p < 0.001), and a significant interaction of group by ROI (F(6.8,121.5) = 2.79, p = 0.011). To follow up this significant interaction, we performed independent sample t-tests. We found that the 2^nd^ ROI along the crown of the gyrus (corresponding approximately to 1.34 degrees eccentricity) was significantly thinner in the MD group compared to the control group (T(18) = −2.41, p = 0.03). The 5^th^ crown of the gyrus ROI (corresponding approximately to 7.3 degrees eccentricity) was significantly thicker in the MD group compared to the control group (T(18) = 2.58, p = 0.02). No other post hoc t-test with this set of ROIs was significant (Fig. 3B).

#### Circle ROI - bank of the sulcus

We performed a two-way mixed-measures ANOVA on data from the bank of the sulcus ROIs (from Fig. 2D) with a between-subjects factor of group (2 levels) and within-subjects factor of ROI (9 levels). These data violated the repeated measures ANOVA assumption of sphericity with a Huynh-Feldt Epsilon = 0.86. The data are reported with the corrected degrees of freedom. There was no main effect of group (F(1,18) = 0.216, p = 0.648), but there was a main effect of ROI (F(6.8,123.1) = 15.72, p < 0.001). There was no significant interaction of group by ROI (F(6.8,123.1) = 1.251, p = 0.28) (Fig. 3C).

#### Circle ROI -depth of the sulcus

We performed a two-way mixed-measures ANOVA on data from the depth of the sulcus ROIs (from Fig. 2C, Magenta colored regions) with a between-subjects factor of group (2 levels) and within-subjects factor of ROI (9 levels). These data violated the repeated measures ANOVA assumption of sphericity with a Huynh-Feldt Epsilon = 0.84. The data are reported with the corrected degrees of freedom. There was no main effect of group (F(1,18) = 1.32, p = 0.27), but there was a main effect of ROI (F(6.7,121.2) = 5.24, p < 0.001). There was no significant interaction of group by ROI (F(6.7,121.2) = 1.10, p = 0.37) (Fig. 3D).

### Cortical thickness near the scotoma border

**Figure 4:**
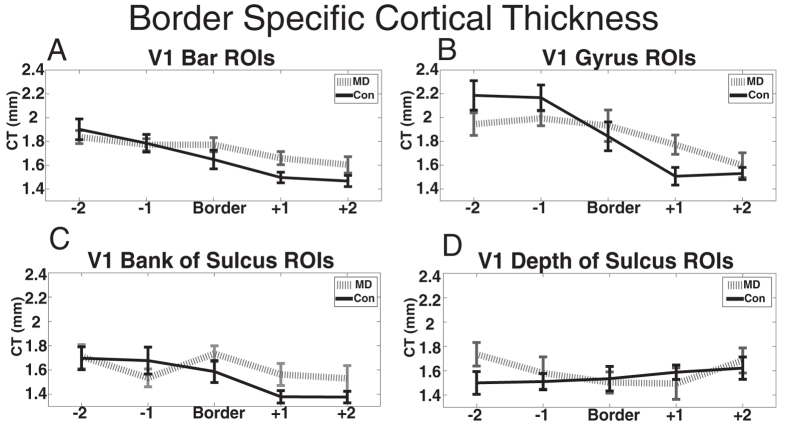
Cortical thickness of macular degeneration (MD) and Control participants in the same sets of ROIs as in Fig. 3. The data are the same as in Fig. 3, but each participant’s data are aligned based on the eccentricity of that participant’s scotoma border. ROIs are: (**A**) Bar ROIs as shown in Fig. 2B (**B**) Gyrus Peak Circle ROIs shown in Fig. 2C (**C**) bank of the calcarine sulcus Circle ROIs shown in Fig. 2D (**D**) depth of the calcarine sulcus Circle ROIs shown in Fig. 2C. Error bars show standard error of the mean.

In order to investigate how changes in cortical thickness relate to the border between the scotoma and the start of spared retinal tissue, we performed a series of analyses in which we took into account the location of each participant’s scotoma border. The participants with central vision loss had a range of scotoma sizes from 8 to 14 degrees in diameter. This corresponds to a range of eccentricities of 4–7 degrees, as these scotomas were centered around the fovea. Based on these eccentricities, the V1 cortical representation of this scotoma border lay in either the 4^th^ or 5^th^ ROI in each MD participant. In order to examine how cortical thickness changed relative to the scotoma border, we aligned the same data from Fig. 3 to the scotoma border ROI for each subject and their matched control. We included 5 regions in total for this analysis: the two closest centrally responsive regions (Border −2, Border −1), the border region, and the two closest peripherally responsive regions (Border +1, Border +2). For each set of ROIs in Fig. 2 we conducted a two-way mixed measures ANOVA with factors of group (2 levels) and ROI (9 levels). The results are presented in Fig. 4.

### Bar ROIs aligned based on the scotoma border

We performed a two-way mixed-measures ANOVA on data from the bar ROIs with a between-subjects factor of group (2 levels) and a within-subjects factor of ROI (ROIs from Fig. 2B; 9 levels) on data from regions aligned at the scotoma border. These data violated the repeated measures ANOVA assumption of sphericity with a Greenhouse-Geisser Epsilon of 0.57 (Huynh-Feldt Epsilon = 0.69). The data are reported with the more strict Greenhouse-Geisser corrected degrees of freedom, as the Huynh-Feldt Epsilon was under 0.75^27^. There was no main effect of group (F(1,4) = 0.24, p = 0.63), but there was a main effect of ROI (F(2.3,40.7) = 20.04, p < 0.001) and a non-significant interaction of group by ROI when corrected for sphericity violations (F(2.3,40.7) = 2.67, p = 0.075) (Fig. 4A).

### Crown of gyrus circle ROIs aligned based on the scotoma border

We performed a two-way mixed-measures ANOVA on data from the crown of the gyrus ROIs (from Fig. 2C, blue) aligned to the scotoma border with a between-subjects factor of group (2 levels) and within-subjects factor of ROI (9 levels). These data violated the repeated measures ANOVA assumption of sphericity with a Huynh-Feldt Epsilon = 0.85. The data are reported with the corrected degrees of freedom. There was no main effect of group (F(1,4) = 0.00, p = 0.96), but there was a main effect of ROI (F(3.4,61.0) = 16.87, p < 0.001) and a significant interaction of group and ROI (F(3.4,61.0) = 3.20, p = 0.03). To follow up this significant interaction, we performed independent sample t-tests. The peripheral region directly adjacent to the scotoma border (Border + 1) was significantly thicker in the MD group compared to the control group (T(18) = 2.42, p = 0.03). No other post hoc t-test with this set of ROIs was significant (Fig. 4B).

### Bank of the sulcus circle ROIs aligned based on the scotoma border

We performed a two-way mixed-measures ANOVA on data from the bank of the sulcus ROIs aligned to the scotoma border with a between factor of group (2 levels) and within factor of ROI (ROIs from Fig. 2D; 9 levels). These data violated the repeated measures ANOVA assumption of sphericity with a Huynh-Feldt Epsilon = 0.80. The data are reported with the corrected degrees of freedom. There was no main effect of group (F (1,4) = 0.76, p = 0.40), but there was a main effect of ROI (F(3.2,57.3) = 5.03, p = 0.003). There was no significant interaction of group and ROI (F(3.2,57.3) = 1.85, p = 0.14) (Fig. 4C).

### Depth of the sulcus circle ROIs aligned based on the scotoma border

We performed a two-way mixed-measures ANOVA on data from the depth of the sulcus ROIs (from Fig. 2C, magenta) aligned to the scotoma border with a between-subjects factor of group (2 levels) and within-subjects factor of ROI (9 levels). These data did not violate the repeated measures ANOVA assumption of sphericity. There was no main effect of group (F(1,18) = 0.32, p = 0.58), no main effect of ROI (F(4,72) = 0.86, p = 0.49), and no significant interaction of group and ROI (F(4,72) = 1.0, p = 0.4) (Fig. 4D).

### Grey matter volume

**Figure 5:**
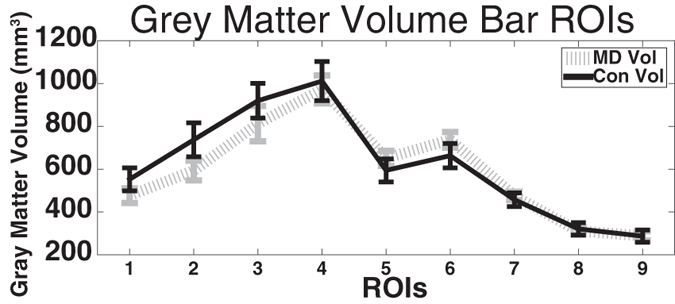
Grey matter volume of primary visual cortex in macular degeneration (MD) and Control participants using the ROIs shown in Fig. 2B. Error bars show standard error of the mean.

We performed a two-way mixed-measures ANOVA with a between-subjects factor of group and a within-subjects factor of ROI (ROIs from Fig. 2B). These data violated the repeated measures ANOVA assumption of sphericity with a Greenhouse-Geisser Epsilon of 0.35 (Huynh-Feldt Epsilon = 0.44). The data are reported with the more strict Greenhouse-Geisser corrected degrees of freedom, as the Huynh-Feldt Epsilon was under 0.75^27^. There was no main effect of group (F(1,18) = 0.22, p = 0.65), but there was a main effect of ROI (F(2.8,50.4) = 72.20, p < 0.001) and there was a non-significant interaction of group and ROI (F(2.8,50.4) = 1.75, p = 0.17). Although not significant, Fig. 5 shows that MD participants exhibited decreased grey matter volume in centrally responsive V1, consistent with the significant interaction of group by ROI. This is in line with previous work using grey matter volume^13^,^15^, and provides further support that the participants enrolled in the current study have similar anatomy to participants in previous studies.

## Discussion

To our knowledge, these data are the first to suggest that central vision loss is associated with both *increases* and *decreases* in primary visual cortical thickness, in the same group of participants. As compared to matched controls, participants with MD had thinner cortex in central V1 areas no longer recruited due to retinal loss as well as thicker cortex in peripheral V1 areas corresponding to spared peripheral vision (Figs 3A,B, 4A,B). Regions whose cortical thickness increased or decreased (relative to controls) mirrored the increased or decreased behavioral importance of the corresponding visual field. Further, these data suggest the increase in cortical thickness preferentially occurred near the border between spared retina and damaged retina (Fig. 4A,B).

MD, by definition, leads to impairment of central vision, and decreased reliance on information from that part of the visual field. As a partial compensation, patients may increase their dependence on peripheral vision. In fact, many individuals develop specific “preferred retinal loci” which they learn to use for tasks involving fine scale vision, such as reading^28^. The structural differences we observe here may underlie compensatory improvements in peripheral vision after central vision loss.

This is a novel study that examined structural plasticity in human V1 as a function of eccentricity using surface-based morphometry. Results from this study will be important for researchers aiming to restore loss of vision due to retinal diseases, because vision involves levels of processing beyond the retina. Plasticity in V1 and other cortical areas following central vision loss will need to be understood to better determine how to restore vision. In addition, the strategy used here, segmenting areas of cortex that have typically been treated as homogeneous^29^, can provide valuable insight into disease states, as well as basic anatomical properties of the cortex that have previously been overlooked.

Although we did observe differences in cortical thickness between MD and control groups in some ROIs, several regions were not different between the groups. From Fig. 3, one can observe that cortical thickness did not significantly differ between groups at the 1^st^, 4^th^, and 7^th^ through 9^th^ regions in any of the sets of ROI defined in Fig. 2. The 1^st^ ROI is located at the foveal confluence, a region of cortex that is notoriously difficult to separate into different visual areas (V1, V2, V3)^30^. Therefore, the 1^st^ ROI may have included other visual areas that are not V1. Further, given that it is located at the occipital pole, the geometry of the cortical folds at that location may be different from the rest of the sulcus. Because cortical thickness does change between the sulcus and gyrus, this difference in folding may influence the plasticity of cortical thickness there. The 4^th^ ROI lies near the border between the representations of the scotoma and healthy retina, and therefore represents a transition between lost vision and increased vision recruitment. The 7^th^ through 9^th^ ROIs correspond to regions in the far periphery (with means of 25.5, 40, and 63.3 degrees eccentricity respectively), an area of visual space to which neither control nor MD participants likely frequently use. Thus visual space that is used most differently between the MD and control groups corresponded to the regions with the strongest observed cortical thickness differences. Further evidence for this is found in Fig. 4, which shows that cortical thickness changes are selective for regions nearest to the scotoma border.

It is unclear why differences between groups may be present in the crown of the gyrus, but not the depth of the sulcus. One possibility is that this is due to inhomogeneity of scotoma shape in the retina. On average our MD participants had a vertical scotoma of 9.5 degrees in diameter, and a horizontal scotoma of 12.7 degrees in diameter. The depth of the sulcus corresponds to the horizontal meridian, while the crown of the gyrus corresponds to the vertical meridian. Thus the MD participants were more impaired in the visual field associated with the depth of the sulcus, and this may have contributed to our not finding significantly thicker-than-control cortex in that region. However, another possible explanation is that the depth of the sulcus may be under more rigid anatomical restraints to remain thin due to the patterns of gyrification in the cortex^31^.

Previous studies have investigated how the morphometry of visual cortex changes with experience. Plasticity is maximized if sensory loss occurs prior to the critical period^32^. Studies of the early blind have shown an increase in cortical thickness in occipital cortex compared to both sighted controls and the late blind^33^,^34^,^35^. However, our data suggest that the loss of a specific portion of the visual field causes eccentricity-specific morphometric changes in V1.

Previous research into anatomical changes associated with central vision loss have reported decreased grey matter density and decreased grey matter volume in typically centrally responsive V1^13^,^15^, consistent with the grey matter volume analysis shown in Fig. 5. These results are also consistent with presented data in Fig. 3, which demonstrated that central visual cortex is thicker in control than MD patients. Previous studies using grey matter density and volume measures, as in our data in Fig. 5, did not observe an increase in grey matter density in peripherally responsive V1. The discrepancy between the cortical thickness effects observed in cortical areas outside the scotoma boarder (Figs 3 and 4), compared to the lack of an effect while using grey matter volume measurements (Fig. 5, and previous data) is likely due to the fact that cortical thickness assesses distinct aspects of cortical morphometry compared to grey matter density/volume measurements^16^. Thus our use of a cortical thickness metric, coupled with our very detailed ROI approach of examining all of V1, have made our study more sensitive than previous studies to anatomical differences as a function of eccentricity.

It remains unclear what cellular mechanisms might underlie the differences in cortical thickness reported. One possibility is that a change in the long-range connections into V1, as a result of central vision loss, result in changes in cortical thickness. There is evidence for eccentricity-dependent effects of attention in V1^36^. These attentional inputs might be modified after increased or decreased use of portions of the visual field. Future studies should test this hypothesis through the use of diffusion-weighted imaging or functional connectivity MRI in humans or tract tracing studies in foveate primates.

Cortical thickness modifications in V1 may also be driven by mechanisms at the local circuit level. Animal models have shown a decrease in interneuron axon density following binocular foveal lesions in the lesion projection zone in V1^11^. The decreased thickness reported here, and decreased volume previously reported^13^,^14^,^15^, might reflect this decreased interneuron axonal density. Animal models have also shown an increase in horizontal projections occurring at the border of the lesion projection zone for both excitatory and inhibitory neurons^11^,^12^. Further, central and peripheral V1 might also have different cellular architecture. Central V1 has higher cellular and neuronal densities than any other part of non-human primate cortex, including peripheral V1^37^. Dendritic structure of interneurons also varies as a function of eccentricity in V1^38^. The loss of central vision in participants with MD and the resulting compensatory visual strategies may have an impact on cellular structure and organization in V1. Future studies should apply postmortem work on humans or use animal models to investigate the relationship between cortical thickness changes in V1 and underlying cellular structure.

Data from the current study show a significant decrease in cortical thickness from centrally responsive to peripherally responsive regions of V1 in our normally sighted controls. These data are consistent with the left portion of Fig. 2A in a recent report^39^, which does not focus on or statistically test this result. These findings suggest a structural difference between centrally and peripherally responsive regions of V1. More work is needed to investigate this basic anatomical finding in V1.

The present study had several limitations. We did not calculate individual subjects’ retinotopy in this experiment. Retinotopy is difficult to identify in patients with MD, but is possible^40^. However, retinotopy of early visual areas, especially V1, are stereotyped, so anatomy is an excellent predictor of retinotopy^25^; therefore, we feel confident that our estimates of retinotopy are generally appropriate. The current work is somewhat limited by the cross sectional design, although we closely matched the participants on gender, education, and age. A longitudinal study would be necessary to confirm that visual experience caused (rather than is correlated with) the differences in cortical thickness we observed. However, a longitudinal study would be extremely difficult to perform, as it would require identifying MD participants who will eventually develop complete bilateral central scotoma early in their disease. Future work should examine the causal relationship between changes in vision and V1 cortical thickness. Finally, the present study included a limited number of participants, due to the fact that we have very selective inclusion criteria. We are studying only a subset of patients with macular degeneration: to be eligible for this analysis, subjects must have a central scotoma in both eyes, which rules out many possible participants with partial macular vision in at least one eye. Obtaining data from such a select group is difficult and time consuming, and required a strong relationship with our colleagues who see patients. Future work examining larger populations, perhaps shared data across sites, may provide power enough to tease apart possible differences between different forms of MD.

To facilitate other researchers’ contributions to examining these types of questions in their own populations, we have made the Matlab code and the regions of interest that were used in this analysis available online at http://labs.uab.edu/visscher/resources/software-protocols. These regions of interest are useful to anyone studying V1, as they span central to peripheral vision, and have been used in previously published work^41^.

To conclude, these results suggest use-dependent modifications in V1 following central vision loss: both increases in cortical thickness following increased use, and decreases in cortical thickness following decreased use. Compensatory visual strategies (e.g., peripheral vision use) in patients with MD may contribute to cortical modifications in V1. These findings are important to the neuroscience and neuroplasticity fields, as they imply that robust changes in cortical thickness, *both increases and decreases,* are possible in the adult brain. This work improves our understanding of the scope of adult neuroplasticity.

## Additional Information

**How to cite this article**: Burge, W. K. *et al*. Cortical thickness in human V1 associated with central vision loss. *Sci. Rep.* **6**, 23268; doi: 10.1038/srep23268 (2016).

## Figures and Tables

**Table 1:** Demographic data for macular degeneration participants.

Gender	Age (years)	OD	OS	OD Scotoma	OS Scotoma	Education (years)
F	57	20/277	20/290	8.12	8.10	16
F	69	20/63	20/481	6.97	6.57	18
F	81	20/36	20/348	10.21	9.46	17
M	77	20/481	20/63	>31.25	14.28	3
M	58	20/220	20/210	14.77	15.74	16
F	53	20/250	20/200	14.47	13.00	13
F	57	20/265	20/290	14.49	12.43	13
F	66	20/50	20/440	9.86	6.24	17
M	34	20/152	20/175	8.06	7.08	17
M	79	20/58	20/253	16.41	12.20	18

OD = Right eye acuity, OS = Left eye acuity, OD Scotoma = Diameter of the Scotoma on the right eye in degrees eccentricity, OS Scotoma = Diameter of scotoma on the left eye in degrees eccentricity, Education = the last completed year of education for each participant.
